# Post-Translational Modifications That Drive Prostate Cancer Progression

**DOI:** 10.3390/biom11020247

**Published:** 2021-02-09

**Authors:** Ivana Samaržija

**Affiliations:** Laboratory for Epigenomics, Division of Molecular Medicine, Ruđer Bošković Institute, 10000 Zagreb, Croatia; Ivana.Samarzija@irb.hr

**Keywords:** prostate cancer, post-translational modification, phosphorylation, glycosylation, ubiquitination, SUMOylation, acetylation, lipidation

## Abstract

While a protein primary structure is determined by genetic code, its specific functional form is mostly achieved in a dynamic interplay that includes actions of many enzymes involved in post-translational modifications. This versatile repertoire is widely used by cells to direct their response to external stimuli, regulate transcription and protein localization and to keep proteostasis. Herein, post-translational modifications with evident potency to drive prostate cancer are explored. A comprehensive list of proteome-wide and single protein post-translational modifications and their involvement in phenotypic outcomes is presented. Specifically, the data on phosphorylation, glycosylation, ubiquitination, SUMOylation, acetylation, and lipidation in prostate cancer and the enzymes involved are collected. This type of knowledge is especially valuable in cases when cancer cells do not differ in the expression or mutational status of a protein, but its differential activity is regulated on the level of post-translational modifications. Since their driving roles in prostate cancer, post-translational modifications are widely studied in attempts to advance prostate cancer treatment. Current strategies that exploit the potential of post-translational modifications in prostate cancer therapy are presented.

## 1. Introduction

Prostate cancer is the second most commonly occurring cancer in men and the fifth leading cause of death worldwide [1]. Since the actions of androgens and androgen receptor (AR) are among the drivers of prostate cancer [2], one of the therapeutical approaches to treat prostate cancer is androgen deprivation therapy (ADT) and the downregulation of AR signaling, which is accomplished by various strategies [3]. Although this approach brings results in the initial phases of treatment, resistance to therapy develops in a substantial part of patients and they progress to castration-resistant prostate cancer (CRPC). Consequently, novel strategies are needed to supplement actions of ADT. Another widely studied driver of prostate cancer is the dysregulation of the PTEN/PI3K/AKT/mTOR signaling that is frequently target of epigenetic and post-translational modifications as well as genetic alterations in prostate cancer. This axis is involved in every aspect of prostate cancer biology, from cancer cell growth to survival and therapy resistance [4,5,6]. The Janus kinase (JAK)/signal transducer and activator of transcription (STAT) pathway that is mediating the actions of cytokines, interferons, and growth factors is also critically involved in prostate cancer growth and progression [7]. Specifically, the actions of an IL6/STAT3 axis are recognized as a major regulator of prostate cancer progression while the STAT5a/b plays a role in cell viability and growth, DNA repair, epithelial-to-mesenchymal transition (EMT), metastatic dissemination in preclinical models, and resistance to enzalutamide [7]. Other strong inducers of prostate cancer progression include EGFR family signaling pathways that are, among other downstream targets, often converging to mitogen-activated protein kinases (MAPK) to regulate prostate cancer cell behaviors [8]. Additionally, hypoxia-induced signaling has been shown to promote prostate cancer progression [9].

In comparison to other cancer types, prostate cancer displays lower frequency of mutations and other genetic alterations. However, frequent genetic changes in prostate cancer include formation of a TMPRSS2/ERG fusion protein, AR amplifications, PTEN deletions and p53 mutations [10]. Other frequent genetic alterations in a subgroup of prostate cancer patients are the mutations in the E3 ubiquitin ligase adapter SPOP, which is found early during prostate cancer development [10].

Proteins and pathways listed above as driving forces of prostate cancer are the subjects (or executors, such as SPOP) of post-translational modifications (PTMs) that are described in further chapters. In this publication, the data from proteome and single protein studies are presented to get an overview of documented PTMs in prostate cancer that influence largely its biology. Additionally, the possibilities of therapies that target PTMs in prostate cancer are discussed.

## 2. Post-Translational Modifications

The biological systems are characterized by extreme complexity that is sustained by concerted actions of plentitude of players. Proteins are among the main biomolecules that perform wide spectrum of diverse functions including signaling, transport, biochemical reactions, and structural support. After translation is complete, majority of proteins undergo different ranges of post-translational modifications (PTMs), chemical modifications that influence largely their activity and functional abilities. The most common PTMs include phosphorylation, glycosylation, ubiquitination, SUMOylation, acetylation, and lipidation [11]. These processes are executed and fine-tuned by thousands of enzymes whose actions are tightly regulated. Deregulations in steps of PTMs leads to pathologies and this is very well documented in different types of cancers were virtually every process in the cell and the main drivers, from tumor suppressors and oncogenes to transcription factors and signaling molecules, are influenced by PTMs [12,13,14,15]. Consequently, PTMs provide potential sites of intervention where tumor promoting events could be suppressed as a part of anticancer therapy [12,14,15,16].

An increasing number of proteome-wide studies of PTMs offer insight into global changes in PTMs in different experimental settings. More than 300 different types of protein PTMs have been described, but only a small proportion have been investigated at the proteome level [17]. PTMs increase the proteome size from thousands to the order of millions of possible protein forms [18], indicating the complexity of the tasks of analyzing PTMs globally. The proteome-wide approach to study PTMs is especially valuable and additive in cases where protein expression levels and mutational status do not differ between conditions that are analyzed, but the changes in the function or the activity of proteins are determined on the levels of PTMs. This paper reviews evidences on the example of prostate cancer, that the tumor driving events are detectable and dissectible by proteome-wide PTM analysis. Findings from proteome studies that encompass changes in phosphorylation, glycosylation, ubiquitination, SUMOylation, and palmitoylation in different experimental settings in prostate cancer are summarized in Table 1 and discussed further in text. The data from single protein studies are added for a complete picture on PTMs in prostate cancer to emerge.

## 3. Post-Translational Modifications in Prostate Cancer

### 3.1. Phosphorylation

Phosphorylation is one of the most extensively studied and among the most frequently experimentally observed PTMs [11]. It is considered that around 40% to 60% of proteins are temporarily phosphorylated. This PTM is used as a molecular switch for protein activity, which regulates almost every aspect of cellular processes from growth, differentiation, and apoptosis to cell signaling. Phosphorylated proteins or proteins involved in phosphorylation (kinases and phosphatases) are among the main drivers of many cancers including that of prostate. Both AR and the PTEN/PI3K/AKT/mTOR axis are driven by phosphorylation, which directs the outcomes of their actions. AR is phosphorylated at 18 sites by many different enzymes, which influences its stability, nuclear localization, and transcriptional activity [44,45,46], which in turn regulates the prostate cancer cell fate.

**Figure 1:**
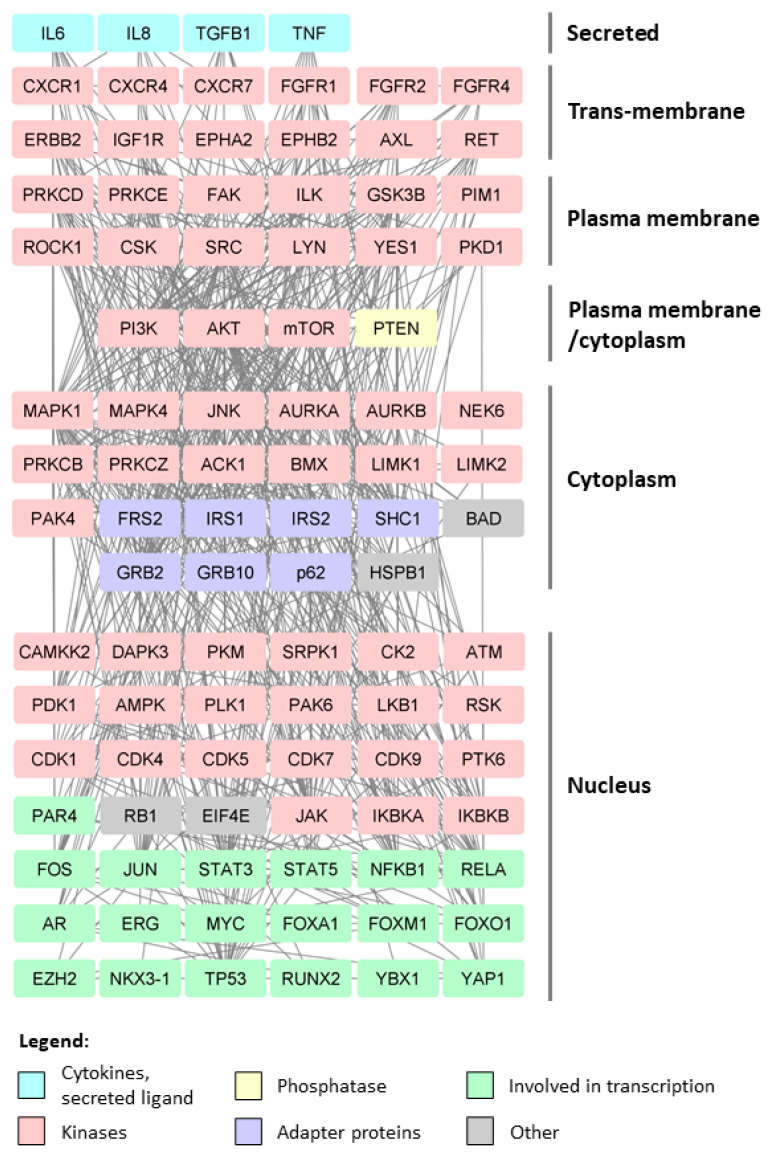
Interaction network of proteins involved in or affected by phosphorylation in prostate cancer. Kinases, adapter proteins, and transcription factors are shown to visualize the network that drives prostate cancer progression. The roles of phosphatases in prostate cancer have been recently reviewed [88] and are omitted from this presentation with the exception of PTEN. Protein–protein interactions were downloaded from the STRING [89] website (experiments and databases interaction sources were used) and visualized in Cytoscape [90]. Proteins were selected based on in vitro and in vivo functional and ex vivo studies.

Phosphatidylinositol-3 kinases (PI3Ks) are a large family of lipid kinases activated mainly by receptor tyrosine kinases. After activation, a wave of downstream signaling events takes place initiated by a synthesis of a lipid secondary messenger phosphatidylinositol 3, 4, 5 trisphosphate (PIP3) to mediate cell growth, proliferation, and apoptosis. The tumor suppressor, phosphatase and tensin homolog deleted on chromosome 10 (PTEN), negatively regulates PI3K signaling by converting PIP3 back to phosphatidylinositol 4,5 bisphosphate. PIP3 recruits phosphoinositide-dependent kinase 1 (PDK1) and AKT to the plasma membrane, where PDK1 phosphorylates AKT at Thr-308. This is further complemented by the action of mammalian target of rapamycin complex 2 (mTORC2), which phosphorylates AKT at Ser-473 for its full activation [5]. Part of this signaling axis is depicted on Figure 1 and involves phosphorylation of downstream AKT targets, such as FOXOs, GSK3, and NF-κB. As seen on Figure 1, functional studies have revealed many kinases to be involved in prostate cancer biology. Growth factor and cytokine signaling conveyed by different receptor kinases (e.g., ErbB2 [47], FGFRs [48,49,50,51,52,53], IGF1R [54,55,56,57,58], CXCRs [59,60,61,62,63,64,65,66,67,68,69]) as well as plasma membrane and cytoplasm located kinases are shown to critically influence prostate cancer. As an example, MAPKs are serine/threonine kinases that link extracellular signals to downstream machinery that influences cell behaviors. Among them, MAPK1 [70,71,72], MAPK4 [73], and JNK [74] show the most prominent roles in regulation of prostate cancer progression. They are shown to activate multiple substrates in response to various stimuli including cytokines, growth factors, oxidative stress, ultraviolet radiation, and drugs. Another potent inducer of prostate cancer cell growth is JAK/STAT signaling axis, which additionally regulates resistance to ADT [75] as well as the immune escape of CRPC to natural killer (NK) cells in hypoxia [76], which could be exploited in immunotherapy against prostate cancer.

Prostate cancer metastasis formation is, among other proteins, influenced by EphA2 [77], steroid receptor coactivator (SRC) family tyrosine kinases [78], FAK [79,80], PKM2 [81], TGFβ [82], TNFα [83], and chemokine signaling at every step of the metastatic process. Specifically, CXCL12/CXCR4 signaling axis in prostate cancer bone metastasis (the main site of prostate cancer dissemination) participates in the formation of the endosteal niche, and blocking this axis compromises initial establishment of tumors in the bone microenvironment. Contrary, the expanding bone tumors are sensitive only to the members of growth factor receptor inhibition [66].

Cytoplasmic adapter proteins that become phosphorylated and activated downstream of many kinases are a link between kinases and other events of signaling cascades. Several adapter proteins (Figure 1) have been shown to influence prostate cancer biology. As an example, FRS2α (FGF receptor substrate 2α), is an FGF receptor-associated protein that was shown to regulate prostate development, regeneration, tumorigenesis and tumor angiogenesis [84,85]. Recently, it was shown that growth factor receptor-bound protein 10 (GRB10), has pro-proliferative function in prostate carcinoma [86], and that it sustains AR activity by interacting with PP2A [87].

The examples from the previous paragraphs show how each step of the tumorigenesis/metastasis formation of prostate cancer is tightly regulated by different cascades that include phosphorylating or phosphorylated proteins. However, although prostate cancer is widely influenced by (de-)phosphorylating enzymes, activating tyrosine kinase mutations or amplifications are very rare in this cancer [91]. Therefore, phosphoproteome studies offer insights in their deregulations. The studies from Drake’s laboratory established that MERTK and NTRK2 kinases drive prostate cancer bone and visceral metastasis [25]. Further, by comparing phosphoproteomes of treatment naive and metastatic CRPC tissue samples, Drake and colleagues revealed critical involvement of proteins from migration and invasion, nuclear receptor, PI3K/AKT/mTOR, stemness, cell cycle, and DNA repair pathways. Moreover, they suggest possible stratification of patients that could benefit from personalized therapy based on their data [22], a strategy that is described in more detail in Chapter 4. Additional proteome-wide studies listed in Table 1 investigated protein phosphorylation in orthotopic xenograft tumors grown in either intact or castrated mice and suggested that YAP1 and PAK2 kinases are involved in androgen-independent cell growth [27]. Another study from a mouse model showed elevated kinase signaling including EGFR, EPHA2, JAK2, ABL1, and SRC tyrosine kinase activation in prostate tumors [24]. Taken together, studies that investigate the roles of single proteins, as well as phosphoproteome studies, complement each other for a complete picture on the deregulation of phosphorylation in prostate cancer and its driving role in prostate cancer biology.

### 3.2. Glycosylation

Glycosylation is the attachment of a carbohydrate (glycan) to functional groups of amino acids. Two main types of glycosylation are recognized: N-glycosylation, when glycans are added to the amide group of an asparagine (Asn) residue in the endoplasmic reticulum and O-glycosylation, when they are added to the hydroxyl oxygen of serine/threonine residues (Ser/Thr) in the Golgi apparatus. Hundreds of enzymes are involved in glycosylation and, unlike other PTMs, attached glycans are extremely diverse and add significantly to the complexity of the final protein structure. Glycome, or the entire complement of sugars, exceeds the complexity of the proteome by the orders of magnitude and nearly every protein rose after the appearance of multicellular life is composed of both polypeptide and glycan parts [92]. Glycosylation is the most common PTM in cells [11] and it is involved in cell adhesion and metastasis, transmitting signals across plasma membrane, and immune modulation [93]. The expression of glyco-genes is mainly regulated on the levels of gene polymorphisms or stable epigenetic regulation [94] that is often dysregulated in cancer [95].

In prostate cancer, glycosylation and the enzymes involved play central roles in tumor progression as shown in Table 2 and reviewed recently [96,97]. Glyco-genes that participate in the formation of both O- and N-linked glycans are deregulated in prostate cancer leading to effects on every aspect of cellular processes and behaviors, from cell proliferation, migration, apoptosis, and viability to in vivo tumor growth and metastasis formation (Table 2). Sialylation, or the addition of sialic acid residues as the terminal monosaccharide, is a process that is disturbed in cancers [93,98]. Sialylated blood group antigen Sialyl Lewis X (SLeX) influences prostate cancer progression through various mechanisms as well as the cancer-associated sialyl-Tn glycan (sTn), which affects prostate cancer cell adhesion and whose expression is shown to be regulated by AR [99]. Fucosylation is the addition of a fucose to a glycan, as either a terminal glycan or the addition to the core structure. As seen on Table 2, fucosylation is dysregulated in prostate cancer progression and participates in the development of castrate resistance. The addition of O-GlcNAc to proteins (O-GlcNAcylation) is catalyzed by O-GlcNAc transferase (OGT) whose substrate, UDP-GlcNAc, is synthesized in the hexosamine biosynthetic pathway (HBP). Inhibition of the HBP was also shown to promote CRPC [100]. OGT was shown to be an essential mediator in androgen-independency also in a glycoproteomic approach where O-GlcNAc chromatin consensus motif imposed by OGT was used as a bait for MS and complemented with MYC ChIP-MS in PCa cells [33]. In this way, it was shown that high OGT activity is essential for proliferation of prostate cancer cell that is driven by MYC. The glycoproteomic approach to prostate cancer yielded several other publications (Table 1) that show the diversity of processes in prostate cancer that implicate glycosylation.

Besides the steps of glycosylation processes that are dysregulated in prostate cancer, and that affect multiple proteins globally, single glycosylated proteins or the proteins that bind glycans have been implicated in prostate cancer. Proteoglycans are proteins that are extensively glycosylated. Among them, versican, decorin, biglycan, lumican, and syndecan-1 were shown to influence prostate cancer cell survival and metastasis [96,116]. Galectins are glycan binding proteins widely studied in the field of prostate cancer research [117] and, among them, galectin-3 was linked to tumor progression [118] and bone remodeling of the bone metastasis niche [119].

In addition to playing driving roles in prostate cancer, another important feature that glycosylation brings to the field of prostate cancer research is the potential to serve as a source of biomarkers of disease progression and severity. One of the central issues in prostate cancer management is to distinguish indolent and aggressive prostate cancer to tailor the treatments accordingly. Current methods that are available do not offer satisfying solution to this issue. Therefore, glycans were suggested to be considered as a supplement to the current tools to diagnose prostate cancer rapidly, and to precisely determine tumor aggressiveness and prognosis [96]. As an example, sialylation of the prostate-specific antigen (PSA), a serine protease secreted by prostate that liquefies semen, has shown the robust prediction power to diagnose aggressive prostate cancer [120,121].

### 3.3. Ubiquitination

Ubiquitination (or ubiquitylation) is a covalent attachment of a ubiquitin, a small protein composed of 76 amino acids (8.5 kDa) with highly conserved primary sequence to lysines of a substrate protein via an isopeptide bond. Depending on the number of ubiquitin units attached, proteins can be monoubiquitinated (addition of a single ubiquitin molecule) or polyubiquitinated (sequential addition of more ubiquitin molecules to preceding ubiquitins). The polyubiquitin chains are named, according to which of the seven lysines (K) within ubiquitin are used to link the chains. Ubiquitination is a process that controls protein abundance, function, and trafficking. Usually, K48-linked chains lead to degradation of a substrate, while K63-linked chains can alter the protein’s activity, interaction, or localization. Furthermore, monoubiquitination mainly plays a role in protein trafficking while polyubiquitination, in addition to protein trafficking, contributes to degradation. Proteins modified by ubiquitin addition are recognized by the proteasome, a cylinder-shaped multi-protein cellular structure located in the cytoplasm and the cell nucleus, which cleaves and degrades or modulates the proteins. This system is known as the ubiquitin-proteasome system (UPS) and its precise control ensures that unnecessary, damaged, misfolded, and potentially harmful proteins are removed. Virtually every process in the cell, from the cell cycle to cell adhesion, migration, invasion, apoptosis, differentiation, angiogenesis, and tumor growth, antigen processing, cytokine signaling, transcription, and DNA damage response is regulated by the UPS [122,123]. Many proteins are involved in the processes of ubiquitination, which is divided in three steps. In a first step, ubiquitin is activated by the ubiquitin-activating enzyme (E1) via using ATP. Subsequently, it becomes transferred to the ubiquitin-conjugating enzyme (E2) and recruited into the E3 ligase, which binds the substrate protein and targets it for proteasome degradation. It is widely accepted that E3 ligases direct the specificity of the complex towards the substrate, which is ensured by the increase in the number of enzymes from E1 to E3. While there are only two E1 and 38 E2 enzymes, there are more than 600 human E3 ubiquitin ligases, which are classified according to the protein sequence homology into the largest Really Interesting New Gene (RING) finger family with more than 600 predicted members and the HECT (Homologous to the E6-AP Carboxyl Terminus) family, with approximately 30 members in the human genome [124]. There are also less common Ring-Between-Ring (RBR) family [125] with 12 members as well as the U-box type E3 ligases, which create an E2 binding surface that resembles a RING finger [126]. These enzyme families differ also by a mechanism of ubiquitination; while RING finger or U-box E3 ligases act as a scaffold, HECT, and RBR type E3 ligases form transient thioester linkages with ubiquitin before transferring it to the protein substrate. Furthermore, RING type E3 ligases can be classified as single or multisubunit E3 ubiquitin ligases. Example of a multisubunit E3 ubiquitin ligase are the SCF cullin–RING ligases (CRLs). CRLs are the major group of RING-type E3 ligases and SCFs are SKP1-CUL1-F-box protein E3 ligases with 69 different complexes found in humans. U-box type ubiquitin ligases are also classified as E4 ubiquitin ligases, a new class of ubiquitination enzymes [127].

The concerted action of ubiquitination enzymes that leads to control of protein abundance and function in a cell is supplemented by the actions of deubiquitinases (DUBs), which remove ubiquitin chains from a substrate and protect it from degradation. This type of interplay between ubiquitinating and de-ubiquitinating enzymes is required to keep the cellular homeostasis [128]. It is estimated that the human genome encodes approximately 100 DUBs [129].

Considering the involvement of ubiquitination in many cellular processes, it is not surprising that the failure to keep it tightly regulated contributes to many pathologies including prostate cancer. The most prominent role among the enzymes involved in ubiquitination in prostate cancer has been described for speckle-type poxvirus and zinc finger (POZ) protein (SPOP) that functions as a substrate adaptor of a CRL3. This protein is altered genetically or through changes in expression in a number of cancers [130]. For prostate cancer, the number of cases with genetic alterations ranges from 4.4% to 28.6% of the cases studied and the number of the cases with downregulation ranges from 25.2 to 93.5% [130]. SPOP mutations are often located in the substrate-binding domain suggesting its biological role. While the role of SPOP protein differs depending on the tumor type, in prostate cancer, SPOP seems to function as a tumor suppressor and its targets for degradation, among others, include AR, ERG, steroid receptor coactivator 3 (SRC3), BRD4, MYC, and TRIM24 [130,131]. The essential role of SPOP in prostate carcinogenesis has been confirmed in different mouse models where SPOP mutations activate the PI3K/mTOR pathway and promote carcinogenesis [132]. Ubiquitylome analysis of a landscape induced by prostate cancer–associated mutations of SPOP in immortalized prostate epithelial cells expressing endogenous SPOP revealed DEK and TRIM24 as substrates consistently upregulated by SPOP mutants and with decreases in ubiquitination and proteasomal degradation (Table 1) [38]. Of these, DEK stabilization was shown to promote prostate epithelial cell invasion [38]. Other proteome studies revealed clusterin as a novel target of E6AP with roles in cell growth [37] and XPC, as a critical mediator of the USP22-mediated response to genotoxic insult [36] (Table 1).

While the roles of DUBs in prostate cancer have been recently reviewed [129], here, in Table 3, the summary on the roles of E3 ligases and the processes affected by their actions are presented. The target proteins of E3 ligases in prostate cancer, among others, are the driving proteins, such as AR, ERG, PTEN, cell-cycle progression proteins, and other transcription factors and signaling molecules (Table 3). According to the current knowledge, AR is ubiquitinated by the actions of RNF6, MDM2, CHIP, and SPOP ligases/ligase subunits, which regulate its stability, transcriptional activity, recruitment of co-activators, chromatin retention, and degradation (reviewed in [133,134]) (Table 3). In addition, the actions of BMI1 affect MDM2-mediated AR protein degradation [135]. SIAH2, an E3 RING finger ubiquitin ligase that influences formation of neuroendocrine phenotype and neuroendocrine prostate tumors through its actions on HIF-1α and FOXA2 [136], is also involved in AR ubiquitination to regulate its transcriptional activity by targeting for degradation a select pool of NCOR1-bound transcriptionally-inactive AR on a group of gene promoters/enhancers [137]. In this way, the subsequent recruitment of AR/coactivator complexes to increase the transcriptional output of selective AR target genes is potentiated. It is interesting to note that in the same publication, the authors did not observe changes in the global levels of AR, emphasizing the subtle roles of SIAH2 in the control of a specific pool of ARs. In a recent work, Vatapalli et al. [138] have shown that upregulation of MYC-regulated E3 ubiquitin ligases HECTD4 and MYCBP2 promotes AR and MYC degradation that leads to repression of MYC in a negative feed forward manner and regulation of the tumorigenicity of AR-positive prostate cancer cells. Among the most prominent E3 ligases that influence prostate cancer is SKP2, an F-box protein, and a crucial component of the SCF type of E3 ubiquitin ligase complexes. SKP2 has been shown to also affect AR ubiquitination [139], but additionally its targets in prostate cancer include EZH2, p27, JARID1B, DAB2IP, AKT, BRCA2, ATF4, p27, p21, and Twist to regulate various cellular processes (Table 3).

On the example of AR ubiquitination, it is evident that the status and the type (site and the number of units involved) of ubiquitination determine whether upregulation or downregulation of a process (AR transcription activity) will occur. In addition, some ubiquitination proteins, such as SPOP regulate global, while the others, like SIAH2, only specific pools of AR. This emphasizes the versatile role that ubiquitination plays in cellular processes and the importance of its tight regulation.

In addition to E3 ligases, there are several publications exploring the roles of E2 enzymes in prostate cancer. It was shown that ubiquitin conjugating enzyme E2T (UBE2T) exhibits oncogenic properties [259] while genetic ablation of Ube2o (ubiquitin conjugating enzyme E2O) impairs progression of prostate cancer. UBE2O targets AMPKa2 for ubiquitination and degradation and UBE2O blockade inhibits tumorigenesis through AMPKa2 restoration [260]. Furthermore, MYLIP is an E3 ubiquitin-protein ligase whose activity depends on E2 enzymes of the UBE2D family and that was shown to affect AR activity in prostate cancer [172].

In conclusion, prostate cancer biology is widely influenced by the actions of enzymes involved in ubiquitination and the main players that drive prostate carcinogenesis are affected and regulated by the UPS system. Understanding of the so-called ‘‘ubiquitin code’’ [261] of prostate cancer through ubiquitomics would add in attempts to dissect the driving events of this disease.

### 3.4. SUMOylation

SUMOylation is the covalent addition of Small Ubiquitin-related MOdifier (SUMO) proteins of about 12 kDa and 100 amino acids to other proteins via an isopeptide bond between the C terminal carboxyl group on the SUMO protein and the amino group on the lysine of the substrate protein. SUMO proteins have four isoforms (SUMO-1, SUMO-2, SUMO-3, and SUMO-4). Although not highly similar at the level of amino acid sequence, SUMO-1 and ubiquitin are related at the levels of secondary and tertiary structures and, consequently the processes of SUMOylation and ubiquitination are mechanistically similar [123]. The SUMOylation process is also executed in three enzymatic steps: SUMO-activation by the enzyme E1 (such as SUMO-activating enzyme SAE1/2); SUMO-conjugation by the enzyme E2 (UBC9—the only SUMO E2 conjugating enzyme discovered to date and the best-characterized E2 enzyme [262]); and SUMO-ligation by the enzyme E3 (such as PIAS/RANBP2/hPC2). The specificity for the target protein of SUMOylation is thought to reside within E2 enzyme. By the in vitro experiments it was indicated that UBC9 is sufficient for binding to the SUMO moiety and transferring SUMO to substrate proteins, but, recently it was suggested that a specific E3 ligase might be required for efficient SUMOylation in vivo by acting as scaffold and helping to add specificity to the SUMOylation reaction [263]. As indicated above, SUMO E3 ligases are classified into three groups: the protein inhibitor of activated STAT (PIAS) family proteins, the polycomb protein Pc2, and RANBP2 (RAN Binding Protein 2, localized to the nuclear pore complex) [263]. SUMOylation is reversible process and de-SUMOylation is catalyzed by SUMO proteases named Sentrin/SUMO-specific Proteases (SENPs), which cleave the terminal glycine of SUMO, releasing it from its core protein to recycle SUMO molecules. This process keeps the low detectable levels of SUMOylated proteins in cells (5–10% of substrate proteins) [127]. There are six SENP enzymes in mammals, which contain a highly conserved 200 amino acid catalytic domain responsible for the enzymatic activity. Other domains vary between SENPs and play roles in subcellular localization and possibly substrate recognition [263]. On the list of experimentally documented and predicted PTMs, SUMOylation, unlike ubiquitination, is not among the top ten processes [11]. Furthermore, SUMOylation, unlike ubiquitination, does not directly target proteins for degradation but is thought to determine cellular localization and, in some cases, can regulate transcriptional activity or act as a protective mechanism to prevent structural alterations to proteins in response to cellular stress [128,264]. Since quite often the same residues within proteins are targeted by SUMOylation and ubiquitination, SUMOylation may compete with the ubiquitination process [128,264]. Additionally, the processes influenced by SUMOylation include subnuclear structure formation, protein–protein interactions, cellular metabolism, regulation of several intracellular signaling pathways, cell differentiation, cell cycle, DNA damage repair, apoptosis, tissue development, and disease progression [263].

By using quantitative proteomics to identify SUMOylated proteins in SUMO stably transfected PC-3 cells Wen et al. [39] (Table 1) found more than 900 putative target proteins of SUMO. Additionally, they have shown that mutation of newly identified SUMO modification sites of USP39 further promotes the proliferation-enhancing effect of USP39 on prostate cancer cells. Among the SUMOylated proteins in prostate cancer are the driving proteins such as AR [134,263,265,266,267,268,269,270], PTEN [271], p53 [216,272], ATF3 [273], FOXA1 [274], FOXA2 [275], CSR1 [276], TBL1 and TBLR1 [277], WWOX [278], pontin [279] SLUG [280], and SNAIL1 [281]. Two SUMOylation residues (K386 and K520) are defined in AR [134]. The PIAS proteins are reported to act as SUMO-E3 ligases for the SUMO-1 and SUMO-2/3 conjugation to AR in vivo and in vitro [263,265,266]. Recently it was shown that SUMO-3 modification by PIAS1 modulates AR cellular distribution and stability [267]. By using wild type and doubly SUMOylation site mutated AR, Sutinen et al. showed that SUMOylation does not simply repress the AR activity, but it regulates AR’s interaction with the chromatin and the target gene selection [268]. Further, androgen-induced, dynamic SUMOylation is linked to the activity cycles of AR in the cell nucleus and chromatin binding, while the stress-induced SUMO-2/3 modifications sustain the solubility of the AR and protect it from short-term proteotoxic insults such as hyperthermia in the nucleus [269]. Clinically isolated substitutions at a SUMOylation related sites in AR lead to SUMOylation that affects multiple endogenous genes. These alterations in AR SUMOylation play significant roles in AR-based diseases, oligospermia, androgen insensitivity syndrome, and recurrent prostate cancer [270].

Of other proteins critically involved in prostate tumorigenesis, it was shown that SUMO-1 modification of PTEN regulates tumorigenesis by controlling its association with the plasma membrane, which in turn affects PI3K/AKT pathway [271]. Additionally, androgen induces SUMO-mediated p53 nuclear export that promotes treatment-resistant prostate cancer [272]. Pro-invasive properties of prostate cancer cells are also regulated by SUMOylation; it was shown that SNAIL1 is regulated by SUMOylation in response to TGFβ stimulation [281] and that p14ARF stabilizes SLUG through increased SUMOylation at lysine residue 192 [280].

Studies that examined the roles of proteins involved in SUMOylation revealed that PIAS1 is a central factor that influences prostate cancer cell proliferation and survival and tumor growth in vitro and in vivo most probably through increased expression of tumor suppressor p21 and declined expression of anti-apoptotic protein Mcl1. Moreover, the same study suggests that PIAS1 is a valid target in docetaxel resistant cells [282]. The work from Palvimo laboratory revealed that PIAS1 is a chromatin-bound AR coregulator that functions in a target gene selective fashion to regulate prostate cancer cell growth [283]. Recent work from the Culig laboratory established PIAS1 as a positive feedback regulator of AR signaling, which is achieved through enhanced AR stabilization in prostate cancer [284].

SENP1 is reported to be the de-SUMOylation enzyme that cleaves the SUMO-1 group from SUMOylated AR protein and reverses the ligand-induced SUMOylation of AR to help to fine tune the cellular responses to androgens in a target promoter-selective manner [285]. SENP1 also promotes EMT of prostate cancer cells via regulating SMAD4 de-SUMOylation [286] and it regulates PTEN stability to promote prostate cancer development [258]. In a mouse model, Bawa-Khalfe et al. showed that SENP1 overexpression induces transformation of the normal prostate gland and gradually promotes the onset of high-grade prostatic intraepithelial neoplasia through induction of HIF-1α-dependent angiogenesis and increased cell proliferation [287]. Similarly, Wan et al. showed that SENP1 promotes prostate cancer progression and metastasis [288]. As a connection to the ubiquitination pathway in prostate cancer, it was shown that SPOP promotes cellular senescence by degrading the SENP7 [289]. Taken together, these results indicate that investigated SENPs in prostate cancer induce cancer progression and a malignant phenotype, while the work on SUMOylation revealed the frequent modification of prostate cancer driving proteins.

### 3.5. Acetylation

The acetylation of proteins is a dynamic and highly specific PTM in which acetyl donors (such as acetyl-CoA) transfer acetyl groups to the proteins under the catalysis of acetyltransferase. In the opposite reaction, proteins are deacetylated by the actions of deacetylases. Protein acetylation is one of the main regulators of gene transcription since most histone acetyltransferases are located in the nucleus where they act as transcriptional co-activators [290]. Historically, acetylation was first discovered as a process affecting histones and if lysine is acetylated, histones will no longer be positively charged, so the binding of DNA to the histone is relaxed, which facilitates gene transcription. However, currently >100 non-histone proteins that are involved in transcription are shown to be affected by acetylation confirming that regulation of gene transcription is a major role of non-histone protein acetylation too [291]. Protein acetylation is also associated with protein degradation and it can regulate a variety of signaling pathways as well as the cell cycle [292]. To recognize the large amount of non-histone protein acetylation, histone acetyltransferases (HATs) and histone deacetylases (HDACs) were renamed to lysine acetyltransferases (KATs) and lysine deacetylases (KDACs), respectively. The documented roles of these proteins in prostate cancer are summarized in Table 4.

Globally, hypoacetylation and excessive histone deacetylase activity in prostate cancer cells has been observed [293,294] and global histone modification patterns (acetylation and dimethylation in histones H3 and H4) predict progression, development and risk of prostate cancer recurrence [295,296]. These patterns change under chronic hypoxic conditions in the prostate [297]. Additionally, acetylation of the histone variant H2A.Z occurs at active promoters and is associated with oncogene and neo-enhancers activation in prostate cancer [298]. The transition of androgen-dependent to -independent prostate cancer was suggested to be associated with the changes in protein lysine acetylation as the number of cellular proteins undergoing acetylation in the androgen-dependent prostate cancer was higher as compared to the androgen-independent [299]. H3-lysine-27 acetylation (H3K27ac) ChIP-seq in Enzalutamide (Enz)-resistant CRPC cells identified a group of super enhancers that are abnormally activated in Enz-resistant CRPC cells and associated with enhanced transcription of a subset of tumor promoting genes such as CHPT1 that drive anti-androgen therapy resistance in prostate cancer [300]. Takeda et al. showed that the AR candidate enhancer becomes histone acetylated (H3K27ac) in CRPC tumors and that deacetylation of this enhancer element effectively suppresses AR signaling and decreases sensitivity to Enz [301]. Among those listed in Table 4, other proteins that direct AR acetylation include Arrest defective protein 1 (ARD1) that is androgen induced [302] and promotes AR dissociation from HSP90 complex and prostate tumorigenesis [303]. Acetylation of AR enhances coactivator binding [304] and in turn histone acetylation, among other factors, defines genomic AR-occupied regions [305]. Deregulation of AR expression is a driver of chromatin relaxation and AR/androgen-regulated bromodomain-containing proteins (BRDs), which are histone acetylation readers, mediate this effect, which helps to stratify patients with tumors in which BRD-mediated TF binding is enhanced or modified as cancer progresses. Those patients could potentially benefit from combination therapy targeting bromodomains [306].

Of other proteins implicated in prostate cancer, transcriptional regulator and tumor suppressor Id4 was shown to regulate transcriptional activity of wild type and mutant p53 via K373 acetylation in prostate cancer [315,382], which directs selective transcription complex assembly [383]. Mechanistic insights into p53 acetylation come from studies that showed that resveratrol enhances p53 acetylation and apoptosis in prostate cancer by inhibiting MTA1/NuRD complex [384]. It also regulates PTEN/AKT pathway through inhibition of the same complex [385]. Metastasis-associated protein 1 (MTA1) is a part of the nucleosome remodeling deacetylation (NuRD) corepressor complex that mediates posttranslational modifications of histones and non-histone proteins, which leads to transcriptional repression.

The transcription factors Krüppel-like factor 5 and 6 (KLF5 and KLF6) are critically involved in prostate cancer progression [386,387]. Recently, it was shown that KLF5 acetylation regulates luminal differentiation of basal progenitors in prostate development and regeneration [388] and is involved in TGFβ caused docetaxel resistance [389]. Remarkably, the acetylation status of KLF5 determines whether this protein will switch from its tumor suppressor function to tumor promoter in prostate cancer cells [390]. Additionally, different expression patterns of acetylated and unacetylated KLF5 in prostatic epithelial cells have been reported [391]. For KLF6, it was also suggested that acetylation may regulate its function [392]. In conclusion, global as well as single protein aberrant acetylation is critically involved in many aspects of prostate cancer progression.

### 3.6. Lipidation

Protein lipidation that includes cysteine palmitoylation, N-terminal glycine myristoylation and cystein prenylation (farnesylation and geranylgeranylation) is frequently detected in eukaryotic proteins [11] and is involved in membrane trafficking, protein localization and secretion, signal transduction, and apoptosis [393]. In prostate cancer, the palmitoyl-protein signature of extracellular vesicles (EVs) was reported [40] and it was suggested that palmitoylation plays a role in the sorting of the EV-bound secretome. Other reports investigated changes in palmitoylation after androgen treatment [42,43] and DHHC3 palmitoyltransferase ablation [41] (Table 1).

Of the enzymes involved in lipidation, it was shown that fatty acid synthase (FASN), an enzyme that catalyzes de novo synthesis of the fatty acid palmitate, increases prostate cancer cell adhesiveness, impairs HGF-mediated cell migration and reduces three-dimensional (3D) invasion by mediating actin cytoskeletal remodeling downstream of palmitoylated atypical GTPase RHOU [394,395]. Moreover, p63 cell survival promoting capabilities engage the actions of FASN [396] and inhibition of FASN induces endoplasmic reticulum stress in prostate cancer cells [397] as well as down-regulates c-MET expression [398].

Another lipidation enzyme, lysophosphatidylcholine acyltransferase of histone serine palmitoylation (LPCAT1) was found to mediate CRPC growth via nuclear re-localization and histone H4 palmitoylation in an androgen-dependent fashion by increasing mRNA synthesis rates. Additionally, LPCAT1 overexpression led to CRPC cell resistance to treatment with paclitaxel [399].

Farnesyl diphosphate synthase (FDPS), a mevalonate pathway enzyme that synthesizes isoprenoids, plays an oncogenic role in PTEN-deficient prostate cancer progression [400]. Inhibition of FDPS by zoledronic acid reduces growth and clonogenicity of human and murine PCa cells in 2D and 3D by disrupting AKT and ERK signaling through direct interference of small GTPases protein prenylation. Among the numerous products including cholesterol, vitamin K, coenzyme Q10, and all steroid hormones, the mevalonate synthesis pathway produces intermediates for isoprenylation of small GTPases, and it was shown that inhibition of geranylgeranyltransferase (GGTase-I), and farnesyltransferase (FTase) disrupts cytoskeletal organization of human PC-3 prostate cancer cells [401]. Inhibiting geranylgeranyl diphosphate synthesis reduces nuclear AR signaling and progression to neuroendocrine prostate cancer phenotype [402]. The same strategy of targeting protein geranylgeranylation slows tumor development in a murine model of prostate cancer metastasis possibly through reduction in Rap1A geranylgeranylation [403] and reduces adrenal gland tumor burden in a murine model of prostate cancer metastasis [404]. Further to this, statins are a class of inhibitors of 3-hydroxyl3-methylglutaryl coenzyme A (HMG-CoA) reductase, a key enzyme in synthesis of cholesterol. Antitumoral effects of statins in prostate cancer have been attributed to both cholesterol dependent and independent effects. However, the reduced circulating and cellular cholesterol levels are thought to contribute the most [405]. Studies have demonstrated statin therapy to be associated with prostate cancer prevention and favorable clinical outcomes and they suppress tumorigenesis in prostate cancer models [406,407,408,409,410]. In PC-3 cells, statin (atorvastatin, a commonly prescribed statin for treatment of hypercholesterolemia) induces autophagy most likely by inhibiting geranylgeranyl biosynthesis, which suggests that autophagic response to statins may partially underlie the protective effects of statins on prostate cancer progression [411].

Of the individual proteins affected by lipidation, it was shown that alteration of palmitoylation and myristoylation sites change oncogenic potential of constitutively active SRC [412,413,414,415] and FYN kinases in prostate cancer [412]. Moreover, the oncogenic effects of AKT are reinforced by its myristoylation and these effects arise, in part, from the tendency of the membrane-targeted form of the protein to reside in cholesterol-rich membrane microdomains [416]. Palmitoylation of the classical sex steroid receptors is required for membrane localization and function [417] and palmitoylation of KAI1/CD82 is necessary for its inhibitory effect on cell migration and invasion [418]. Furthermore, pharmacologically targeting the myristoylation of the FRS2 scaffold protein whose role in prostate cancer has been elaborated in Chapter 3.1, inhibits FGF/FGFR-mediated oncogenic signaling and consequently the prostate cancer progression [419].

## 4. Therapeutic Potential of Post-Translational Modifications in Prostate Cancer

**Figure 2:**
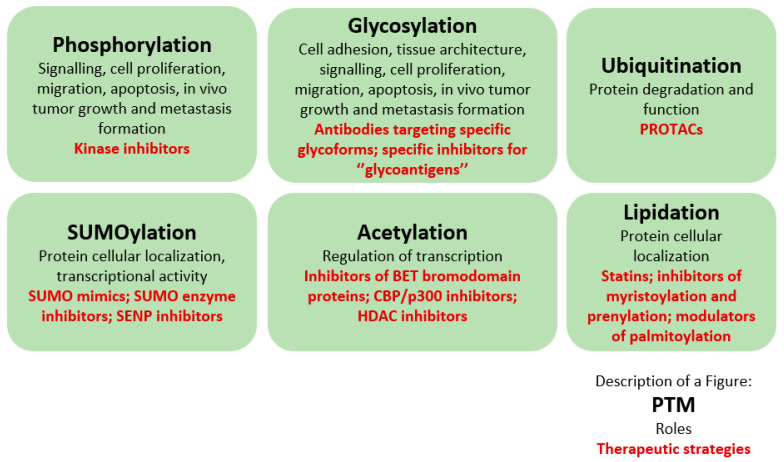
Post-translational modifications, their involvements in cellular processes and therapeutic opportunities.

In previous chapters, critical involvement of PTMs in prostate cancer progression has been documented. As such, targeting PTMs offers the opportunity to interfere with crucial events in cancer biology as depicted on Figure 2.

Since kinases are the driving proteins in both cancer cell growth and dissemination, during the last two decades, several molecules targeting receptor tyrosine kinases were used in oncology as a first or second line therapy in various cancer types [420]. In prostate cancer, tyrosine kinase inhibitors targeting signaling pathways of EGFR, VEGFR, c-SRC family kinases, platelet-derived growth factor and c-MET showed encouraging results in pre-clinical settings but these finding are still waiting to be realized as anticancer drugs as phase III clinical trials did not produce satisfying results [421].

Despite promising results in early preclinical studies, targeting the PI3K-AKT-mTOR pathway in prostate cancer appeared to be challenge due to numerous feedback and feedforward loops and redundancy mechanisms that prevent complete blockage of the pathway [6]. Consequently, combinatorial therapies are explored. Targeting PI3K-AKT-mTOR in combination with AR (phase II study of ipatasertib in combination with abiraterone) showed promising results [422], but further research is needed to delineate the crosstalk between the two pathways and to define biomarkers for patient stratification.

Since the mechanisms of resistance to therapy are expected to vary significantly between patients and in individual tumors, another opportunity for prostate cancer management that is based on phosphorylation emerges from phosphoproteome studies. Analyzing phosphoproteomic profiles of cancer patients would contribute to possible patient stratification and this valuable knowledge could be used in the settings of personalized medicine [423]. Personalized phosphoproteomics or the analysis of signaling networks in individual tumors, advances personalized therapy by discovering biomarkers of pathway activity and therefore, suggesting potential targets [424]. Like mentioned in an introductory part, CRPC often arises in a substantial subgroup of patients worsening the treatment options. Neuroendocrine prostate cancer (NEPC) is an aggressive subtype of prostate cancer with poor prognosis that most commonly arises in later stages of prostate cancer as a mechanism of treatment resistance [425]. Clinically distinct therapeutic strategies are considered against NEPC compared to the AR-driven adenocarcinomas. The studies from Beltran and Drake laboratories established that targeting Aurora A [426] and Ret [427] kinases could bring benefit to NEPC patients. Specifically, a phase II clinical trial of the Aurora kinase A inhibitor alisertib for CRPC and NEPC patients established that in a subset of patients with molecular features supporting Aurora A and N-myc activation significant clinical benefit from single agent alisertib could be achieved [426]. On this example it is evident how precision oncology and disease classification based on genomic sequencing that would consider individual qualities of tumors and the status of the main drivers offers possibilities of improvements of prostate cancer therapy by either providing the targets for the treatment or the biomarkers to guide future pre-clinical research and improve clinical trials [428,429].

The strategy to target oncogenic proteins for degradation is widely used in the attempts to develop novel anti-cancer drugs. A proteolysis targeting chimera (PROTAC) consists of one protein-binding molecule that is capable of engaging an E3 ubiquitin ligase, and another that binds to a target protein meant for degradation. In this way, PROTAC is capable of removing specific unwanted proteins. Recently, the first clinical data were reported for AR PROTAC showing some efficacy and good safety profile in men with metastatic CRPC [430]. Considering that AR is heavily affected by other PTMs [44,45,46,134,431,432], they could potentially be explored in prostate cancer treatment.

SENP [433] and SUMO enzyme inhibitors [434] are studied in the field of prostate cancer and showed efficacy in in vitro studies. While this strategy in prostate cancer still awaits to be further exploited, the (de-)acetylation inhibitors are already used in clinical trials. HDAC inhibitors vorinostat, pracinostat, panobinostat, and romidepsin underwent phase II clinical trials for prostate cancers but results were not satisfying to recommend phase III trials as majority of patients exhibited either toxicity or disease progression [435]. The CBP/p300 bromodomain inhibitor CCS1477, the only CBP/p300 inhibitor currently in clinical trials, is under clinical evaluation for the treatment of prostate cancer [436].

Interference with lipidation showed efficacy in in vivo model where blocking myristoylation of SRC inhibited its kinase activity and suppressed prostate cancer progression [415]. Moreover, statins (inhibitors of cholesterol synthesis) used as a therapy were associated with prostate cancer prevention and favorable clinical outcomes [405].

Although their great importance in cancer progression continues to be shown, glycans are still overlooked in drug discovery strategies, mainly because of the complexity associated with the glycosylation process and technical difficulties in studying this PTM. However, some researchers envision that targeting glycans has the potential to start a new era of cancer therapy especially because glycans are actively involved in tumorigenesis and seem to play a role in the failure of existing cancer treatment options [437]. Glycosylation is recognized as an androgen-regulated process essential for prostate cancer cell viability and is a global target for androgen control suggesting that loss of specific glycosylation enzymes might contribute to tumor regression following ADT [105]. Additionally, as mentioned in Chapter 3.2, global glycosylation profiles or those of individual proteins may serve as a source of biomarkers of disease progression and severity.

Taken together, the driving role of PTMs in prostate cancer biology is widely considered in efforts of finding new drugs targeting this disease with some promising compounds reaching far in (pre)clinical and epidemiological studies of prostate cancer. Besides being the targets for the treatment, PTMs offer potential of biomarkers that could guide future research and therapeutic strategies.

## 5. Conclusions

The main drivers of prostate cancer progression, such as AR [44,45,46,134,431,432], PTEN/PI3K/AKT/mTOR [438], STAT3 [439,440], NKX3.1 [441] are influenced by PTMs, which changes their activity, expression, stability, and localization. Additionally, many enzymes involved in PTMs are deregulated in prostate cancer and this directs prostate cancer cell behaviors. Since their driving role in prostate cancer, PTMs are widely explored in attempts to advance prostate cancer therapy. Proteomics analysis of PTMs in prostate cancer offers valuable information especially in cases when protein expression and/or mutational status do not change in malignancy, but the proteins differ only in the PTMs. Further to this, single protein PTM studies complement proteomics to generate complete catalogue of PTMs in prostate cancer.

## Figures and Tables

**Table 1:** Findings from proteome studies on post-translational modifications that influence prostate cancer cell fate.

PTM	Experimental Setting	Main Findings	Ref.
**Phosphorylation**	Comparative phosphoproteomics of differentially expressed kinases between the highly aggressive PC-3 and PC-3M cells.	PAK2, SLK, MST4, MAP2K2, and ARAF are kinases that are potentially associated with increased migration in PC-3M cells.	[19]
	(Phospho)proteomic profiling of human prostate cancer (PCa)-associated fibroblasts.	PCa-associated fibroblasts-derived LOXL2 is an important mediator of intercellular communication within the prostate tumor microenvironment.	[20]
	Characterization of the ERG-regulated kinome.	TNIK is suggested as a potential therapeutic target.	[21]
	Phosphoproteome of treatment naive and metastatic CRPC tissue samples integrated with genomic and transcriptomic data.	Six major signaling pathways with phosphorylation of several key residues are significantly enriched in CRPC tumors; clinically relevant information (kinase target potential based on patient-specific networks) potentially suitable for patient stratification and targeted therapies in late stage PCa is provided.	[22]
	Analysis of global phosphoproteomic changes induced by fish oil in human PCa.	Pyruvate dehydrogenase alpha 1 is a target of omega-3 polyunsaturated fatty acids in human PCa.	[23]
	Phosphoproteomics data from mouse model of PCa progression [24] integrated with gene expression analysis and literature mining.	A total of 125 wild type kinases implicated in human PCa metastasis were selected for screen for in vivo metastatic ability; the RAF family, MERTK, and NTRK2 kinases drive PCa bone and visceral metastasis, and are highly expressed in human metastatic PCa tissues, potentially representing important therapeutic targets.	[25]
	Comparative phosphoproteome analysis of a PCa cell line, LNCaP, and an LNCaP-derived androgen-independent cell line, LNCaP-AI.	The phosphorylation level of THRAP3 is significantly lower in LNCaP-AI cells; nonphosphorylatable mutant form of THRAP3 and the phosphorylation-mimic form differ significantly in protein binding repertoire; many of the differentially interacting proteins were identified as being involved in RNA splicing and processing.	[26]
	Quantitative proteomic approach to compare protein phosphorylation in orthotopic xenograft tumors grown in either intact or castrated mice.	Changes in phosphorylation of YAP1 and PAK2 and their elevated levels in CRPC identified; YAP2 and PAK2 regulate cell colony formation and invasion in androgen-independent cells; PAK2 influences cell proliferation and mitotic timing; pharmacologic inhibitors of PAK2 and YAP1 are able to inhibit the growth of androgen-independent PC-3 xenografts.	[27]
	Phosphotyrosine peptide enrichment and quantitative mass spectrometry (MS) in oncogene(non-TK)-driven mouse model of PCa progression.	Elevated TK signaling (EGFR, EPHA2, JAK2, ABL1, and steroid receptor coactivator (SRC) tyrosine kinase activation) is recorded.	[24]
	Proteome analysis of Aurora-A substrates using small molecule inhibitor and reverse in-gel kinase assay in PC-3 cells.	NuMA becomes hypo-phosphorylated in vivo upon Aurora-A inhibition; mutation of three of these phospho-sites significantly diminishes cell proliferation and increases the rate of apoptosis; NuMA T1804A mutant mislocalizes to the cytoplasm in interphase nuclei in a punctate pattern.	[28]
	Phosphoproteomics of metastatic docetaxel-resistant PCa cell lines (DU145-Rx and PC-3-Rx).	Increased phosphorylation of FAK mediates chemoresistance in CRPC.	[29]
**Glycosylation**	Proteomics analysis to determine the O-glycan profiles of PCa cells metastasized to bone (PC-3), brain (DU145), lymph node (LNCaP), and vertebra (VCaP) in comparison to immortalized RWPE-1 cells derived from normal prostatic tissue.	PCa cells exhibit an elevation of simple/short O-glycans, with a reduction of complex O-glycans, increased O-glycan sialylation, and decreased fucosylation. Core 1 sialylation is increased in all PCa cells. The expression of sialyl-3T antigen, which is the product of ST3Gal-I is increased. ST3Gal-I is associated with PC-3 cell proliferation, migration and apoptosis. Downregulation of ST3Gal-I reduces the tumor size in xenograft mouse model.	[30]
	Comprehensive proteomic approaches of FUT8 overexpressing PCa cells.	Upregulation of EGFR and its downstream signaling; increased cell survival in androgen-depleted conditions.	[31]
	Extracellular vesicles (EV)-derived glycoproteins upon overexpression of FUT8 in PCa cells.	Reduced number of vesicles secreted by PCa cells; increase in the abundance of proteins associated with cell motility and PCa metastasis; altered glycans on select EV-derived glycoproteins.	[32]
	O-GlcNAc chromatin consensus motif imposed by OGT used as a bait for MS; combination with MYC chromatin immunoprecipitation (ChIP)-MS in PCa cells.	OGT is an essential mediator in androgen-independency, which is the major mechanism of PCa progression.	[33]
	Proteomics of androgen-dependent and androgen-resistant LAPC4 cells.	FUT8 is significantly overexpressed in the androgen-resistant LAPC4 cells; overexpression of FUT8 might be responsible for the decreased PSA expression in prostate cancer specimens.	[34]
	Cell surface Thomsen–Friedenreich (TF) antigen proteome profiling of metastatic PCa cells.	CD44, α2 integrin, β1 integrin, CD49f, CD133, CD59, EphA2, CD138, transferrin receptor and profilin express TF antigen; TF antigen positive prostate cancer cells form significantly more and larger prostaspheres under both non-differentiating and differentiating conditions and express higher levels of stem cell markers.	[35]
**Ubiquitination**	Overexpression or depletion of USP22 in PCa cells and analysis of the ubiquitylome.	Depletion of USP22 sensitizes cells to genotoxic insult; analysis of the USP22-sensitive ubiquitylome identified the nucleotide excision repair protein, XPC, as a critical mediator of the USP22-mediated response to genotoxic insult.	[36]
	Knockdown of E6AP in DU145 cells and analysis of a proteome.	Clusterin is a novel target of E6AP; concomitant knockdown of clusterin and E6AP partially restores cell growth.	[37]
	Changes in the ubiquitin landscape induced by prostate cancer–associated mutations of SPOP in immortalized prostate epithelial cells expressing endogenous SPOP.	DEK and TRIM24 are effector substrates consistently upregulated by SPOP mutants with decreases in ubiquitination and proteasomal degradation resulting from heteromeric complexes of wild type and mutant SPOP protein; DEK stabilization promotes prostate epithelial cell invasion.	[38]
**SUMOylation**	Quantitative proteomics to identify SUMOylated proteins in SUMO stably transfected PC-3 cells.	More than 900 putative target proteins of SUMO are identified; mutation of newly identified SUMO modification sites of USP39 further promotes the proliferation-enhancing effect of USP39 on PCa cells.	[39]
**Palmitoylation**	Palmitoyl-proteomic analysis of large and small cancer-derived PCa EVs [40].	STEAP1, STEAP2, and ABCC4 are identified as PCa-specific palmitoyl-proteins abundant in both EV populations; their localization in EVs is reduced upon inhibition of palmitoylation in the producing cells.	[40]
	Palmitoyl proteomic analysis of breast and PCa cell lines, ±DHHC3 ablation.	Putative substrates include 22–28 antioxidant/redox-regulatory proteins and DHHC3 ablation elevates oxidative stress; DHHC3 ablation, in combination with chemotherapeutic drug treatment, elevates oxidative stress, with a greater than additive effect, and enhances the anti-growth effects of the chemotherapeutic agents; DHHC3 ablation synergizes with PARP inhibitor PJ-34, to decrease cell proliferation and increase oxidative stress.	[41]
	Proteomic experiments using clickable palmitate probe (Alk-C16) between three individual pairs of androgen-treated and non-treated LNCaP cells.	Androgen treatment significantly increased the palmitoylation level of eIF3L, which may be used as a biomarker for the diagnosis of early-stage PCa.	[42]
	LNCaP cells metabolically-labeled with Alk-C16, a palmitate probe and treated with R1881, an androgen, or DMSO after which palmitoylome profiling was performed.	Androgen treatment significantly increases the palmitoylation level of α-tubulin and Ras-related protein Rab-7a (Rab7a), which are essential for cell proliferation; in the supernatant of LNCaP cells, the palmitoylation level of α-tubulin is also increased following androgen treatment, which may represent a biomarker for early-stage PCa.	[43]

**Table 2:** Involvement of glycosylation in prostate cancer biology. The step of glycosylation is stated on the top of each rectangle and examples are schematically depicted. The prostate cancer processes affected are listed on the bottom of rectangles.

**Sialylation** 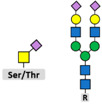 O-linked glycans: in vitro proliferation, migration, apoptosis; tumor size in mouse model [30]; cell adhesion [99]; N-linked glycans: in vitro proliferation, migration, invasion [101].	**Fucosylation** 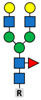 Self-assembly of spheroids [102]; EGFR signaling; cell survival in androgen-depleted conditions [31]; vesicles secreted by PCa cells [32]; PSA expression [34]; metastasis to bone [103].	**Biosynthesis of1,6** **GlcNAc-Branched** **N-glycans** 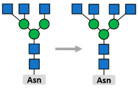 In vitro invasion; tumor growth in xenograft models [104].	**Mannose Trimming of N-glycans** 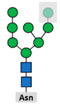 Essential for cell viability [105].
**Regulation of N-glycosylation Substrate Specificity** In vitro proliferation, migration and invasion; xenograft growth in a PTEN negative background; ER structure and stress response; Akt signaling [106].	**O-Linked N-Acetylgalactosamine Addition** 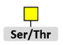 Essential for cell viability [105].	**O-Linked N-Acetylglucosamine Addition** 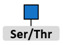 Essential process in androgen-independency [33]; metabolism [107].	**Generation of the Common Core 1 O-glycan Structure** 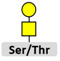 Castration resistance and metastasis [108,109].
**Core-2-branched O-linked glycosylation** 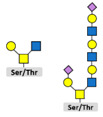 Tumor growth in mouse model [110,111]; cell adhesion [110]; resistance to NK cell immunity [112]; LNCaP susceptibility to apoptosis induced by Galectin-1 [113].	**Core-3 O-linked glycan formation** 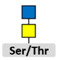 Tumor formation andmetastasis of PC-3 and LNCaP cells through downregulation of α2β1 integrin complex [114].	**I-branching** 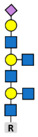 Migration and invasion; integrin signaling via indirect mechanisms; in DU145 cells appears to largely occur on glycolipids and partially on O-glycans [115].	**Legend:** 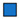 N-acetylglucosamine 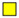 N-acetylgalactosamine 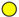 Galactose 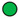 Mannose 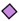 Sialic acid 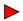 Fucose

**Table 3:** Ubiquitin E3 ligases, their target proteins, and processes they influence in prostate cancer. Signaling pathways regulated by deubiquitinases in prostate cancer have been recently reviewed [129], as well as the roles of the SPOP protein [130,131], substrate adaptor of a CRL3 that is frequently mutated in prostate cancer.

(Component of) E3 Ligase	Description	Affected Protein(s) and/or Signaling Pathways	Effects on Processes
**RING type**			
AMFR	RING-type E3 ubiquitin transferase, component of a complex that participates in the final step of ER-associated degradation	3βHSD1 [140]	DHT synthesis necessary to activate the AR [140]
APC/C	Multi-subunit cullin-RING E3 ubiquitin ligase that regulates progression through the metaphase to anaphase of the cell cycle	Cyclin A2, Geminin, PLK1, Aurora A, and CDC20 [141]; SKP2 [142]	PTEN loss but not phosphatase inactivation results in hypersensitivity to pharmacological inhibition of APC-CDH1 targets PLK1 and Aurora A [141]; cell cycle [142]
BIRC6	Consists of a BIR and a ubiquitin-conjugating (UBC) domain with chimeric E2/E3 ubiquitin ligase activity; through its BIR domain binds to active caspases; through its UBC domain, facilitates proteasomal degradation of pro-apoptotic proteins	GPCR and matrisome signaling; prosurvival genes [143]	Implicated in advanced, Enzalutamide (Enz)-resistant PCa [143]; role in PCa progression and treatment resistance [144]
BMI1	Contains a RING motif; it does not have E3 ubiquitin ligase activities; forms a complex with RING1B to ubiquitinate H2A-K119 and repress the expression levels of polycomb repressive complex 1 (PRC1) targets	AR [135]	PRC1-independent role in MDM2-mediated AR protein degradation; tumor growth of xenografts that have developed resistance to surgical castration and Enz treatment [135]
CAND1	F-box protein exchange factor; key assembly factor of SCF E3 ubiquitin ligase complexes	p21 [145]; PLK4 [146]	In vitro cell viability, proliferation, apoptosis [145]; centriole overduplication [146]
c-CBL	RING domain E3 ligase	EGFR [147]	EGFR/Erk1/2 signaling-mediated PCa [147]
COP1	RING-type E3 ubiquitin transferase	STAT3 [148]; ETS transcription factors [149]	Tumorigenesis; proliferation and cancer stem-like properties in prostate epithelial cells [148,149]
CRL4/Cdt2	Proliferating cell nuclear antigen (PCNA)-dependent E3 ubiquitin ligase	WHSC1 [150]	Interaction with key intracellular signaling molecules, AKT, RICTOR, and Rac1, to drive PCa metastasis [150]
CUL3	Cullin–RING-based E3 ubiquitin ligase		Mutated in a subset of PCa indicating possible driving roles [151]
CUL4A	Cullin family of ubiquitin ligase proteins	AR [152]	AR protein homeostasis [152]
CUL4B	Scaffold protein that assembles the Cullin4B-RING E3 ligase complex	BMI1 [153], c-MYC [154]	Cancer stem-like traits of PCa cells [153]; PCa progression [154]
FBXL2	F-box protein; the receptor subunit of one of 69 human SCF ubiquitin ligase complexes	IP3R3 [155]	Ca2+-mediated apoptosis and tumor growth [155]
FBXL4	Member of the F-box protein family; part of a modular E3 SCF ubiquitin ligase complexes	Potentially ERLEC1 [156]	PCa progression and metastasis [156]
FBXL7	F-box protein that functions as substrate receptor for SCF	c-SRC [157]	Epithelial-to-mesenchymal transition (EMT) and metastasis [157]
FBXO45	Substrate-specific adaptor subunit of SCF E3 ubiquitin ligase complex	PAR4 [158,159]	Cell survival [158,159]; therapy resistance [159]
FBXW7	F-box and WD repeat domain containing 7	AURKA [160]	Pathogenesis of prostatic small cell neuroendocrine carcinoma [160]
FBW7	F-box protein; a substrate receptor for SCF-type E3 ligase	Dual phosphorylated ERG [161]	Driving of prostate oncogenesis [161]
KLHL20	Substrate-binding subunit of Cullin3 ligase	PML, HIF-1α [162]	PCa progression [162]
MARCH5	RING-finger E3 ligase	MCL1 [163]	Apoptosis in response to a BH3 mimetic agent targeting BCLXL [163]
MDM2	The RING domain E3 ubiquitin ligase; key regulator of p53 tumor suppressor protein activity and stability	AR [164,165]; p53 [166,167]; E2F1 [168]; AR-v7 [169]; E-cadherin [170]; activation of p53 and destabilization of AR by combinatorial inhibition of MDM2 and MDMX [171]	Phosphorylation-dependent ubiquitination and degradation of AR by AKT [165]; stem cell integrity [164]; survival and proliferation of genomically unstable tumor cells [167]; prolongs the half-life of the E2F1 protein by inhibiting its ubiquitination (MDM2 displaces SCFSKP2); influences cell proliferation [168]
MYCBP2	Atypical E3 ubiquitin-protein ligase, which mediates ubiquitination of threonine and serine, instead of lysine residues	AR, MYC [138]	Tumorigenicity of AR-positive PCa cells [138]
MYLIP	E3 ubiquitin-protein ligase whose activity depends on E2 enzymes of the UBE2D family	AR [172]	AR activity [172]
PIRH2	Ring finger protein with ubiquitin ligase activity	Epsilon-COP [173]; HDAC1 [174]	Regulation of the secretion of PSA [173]; AR signaling [174]
pVHL	Substrate recognition subunit of the VHL-Elongin B/C E3 ligase complex that targets the HIF-1/2 for proteasomal degradation under normoxia conditions	AR (enhanced AR de-ubiquitination instead of inducing AR ubiquitination) [175]; HIF-1α [176]	Suppression of AR activity [175]; HIF-1 hypoxic response [176]
RNF2	Also known as RING1b or RING2; catalytic subunit of PRC1	TXNIP [177]; CCL2 [178]	Cell cycle arrest and apoptosis [177]; metastasis in mice inoculated intracardially with PC-3M cells [178]
RNF6	RING finger-type E3 ligase	Poly- and mono-ubiquitination of AR [179]	Promotes AR transcriptional activity and specificity [179]
RNF7	RING component of CRL (Cullin-RING ligase)	PHLPP1 and DEPTOR (PI3K/AKT/mTOR axis) [180]; p21, p27, NOXA; ERK1/2 signaling [181]	Proliferation in monolayer and soft agar; clonogenic survival; migration [180]; PCa tumorigenesis [181]
RNF11	RING finger-type E3 ligase	ErbB2 and EGFR [182]	Growth arrest [182]
RNF20 and RNF40	Histone H2B ubiquitin E3 ligases	AR, several cell cycle promoters [183]	Proliferation (due to changed expression of several cell cycle promoters) and modulation of AR transcriptional activity in intact cells [183]
RNF41	Ring Finger Protein 41, E3 ligase	ErbB3 [184]	AR-independent proliferation [184]
RNF126	E3 ligase that contributes to BAG6-mediated quality control	p21 [185]	Proliferation [185]
SIAH2	E3 RING finger ubiquitin ligase; member of the seven in absentia homolog (SIAH) family	EAF2 [186]; AR [137]; AR-V7 [187]; HIF-1α and FOXA2 [136]; Wnt/β-catenin signaling [188]	Apoptosis [186]; lipid metabolism, cell motility, proliferation, cell growth under androgen-deprivation condition in vitro and in vivo, PCa regression upon castration [137]; castration-resistance in PCa therapy [187]; formation of neuroendocrine phenotype and neuroendocrine prostate tumors [136]; inducing and maintaining PCa cells dormancy in bone [188]; death receptor-mediated apoptosis [189]
SKP2	F-box protein; crucial component of the SCF (Skp1-Cullin1-F-box) type of E3 ubiquitin ligase complexes	EZH2 [190]; p27 [191,192,193]; JARID1B [194]; DAB2IP [195]; AKT [196]; BRCA2 [197]; ATF4, p27, p21 [198]; Twist [199]; AR [139]; IDH1/2 [200]; FOXO3 [201]; E-cadherin [202]	TRAF6-mediated ubiquitination of EZH2; progression of PCa and CRPC through upregulation and activation of progenitor genes, as well as AR-target genes [190]; paclitaxel resistance [191]; tumorigenesis [192,193,194,195,196]; proliferation, survival, glucose uptake [196]; homologous recombination and sensitivity to the PARP inhibitor rucaparib [197]; oncogenic-stress-driven senescence [198]; progression and stem cell features of CRPC [199]; cell cycle-dependent metabolic oscillation between glycolysis and TCA cycle [200]; cell migration [202]; high expression is associated with a mesenchymal phenotype and increased tumorigenic potential [203]
SOCS2	Probable substrate recognition component of a SCF-like ECS (Elongin BC-CUL2/5-SOCS-box protein) E3 ubiquitin ligase complex	FLT3 and JAK2 [204]; NDR1 stability; NF-κB transactivation [205]	Metastasis formation [204]; SOCS2-deficiency leads to hyper-activation of NF-κB and downstream pathological implications [205]
TOPORS	RING domain containing E3 ligase	NKX3.1 [206]	Tumor progression [206]
TRAF4	RING domain E3 ubiquitin ligase	TrkA [207]	Metastasis formation [207]
TRAF6	RING domain E3 ubiquitin ligase	p85a [208]; TGFβ type I receptor [209,210]; PS1 [210]; mTOR [211]; AKT [196]; TAK1 [212]; EZH2 [190]	PI3K/AKT signaling; migration [208]; tumor-promoting effects of TGFβ type I receptor [209,210]; activation of mTOR; regulation of autophagy and cell proliferation [211]; proliferation, survival, glucose uptake, in vivo tumor growth [196]; activation of NF-κB signaling downstream of several receptors [212]
TRIM11	E3 ubiquitin-protein ligase; the TRIM motif contains a RING domain		Cell proliferation in vitro and the progression of PCa [213]
TRIM16	It lacks a RING domain found in other TRIM proteins, but can dimerize with other TRIM proteins and has E3 ubiquitin ligase activity	SNAIL signaling pathway [214]	Progression of prostate tumors [214]
TRIM25	RING domain E3 ubiquitin ligase	ERG [215]; G3BP2 [216]	Driving of prostate carcinogenesis [215]; cell growth and survival by modulating p53 signals [216]
β-TrCP	Substrate recognition subunit for the SCFβ-TrCP E3 ligases	HIF-1α [217], Twist [199]; CHD1 [218]; MTSS1 [219]; REST [220]; δ-catenin [221]; AhR [222]; Gli2 [223]	Progression and stem cell features of CRPC [199]; transcription of the pro-tumorigenic TNF–NF-κB gene network [218]; proliferation and migration [219]; AR activity [220]; cell growth [222]
UHRF1	Ubiquitin Like with PHD And Ring Finger Domains 1; E3 ubiquitin ligase		Cell proliferation and biochemical recurrence after radical prostatectomy [224]; epigenetic crosstalk and PCa progression [225]
**RBR type**			
PRKN	Parkin RBR E3 Ubiquitin Protein Ligase		Participates in removal of damaged mitochondria via mitophagy [226]
**U-box type**			
CHIP	U-box type chaperone associated E3 ligase	JMJD1A [227]; SNPH [228]; AR/AR-V7 [229]; AKT signaling pathway [230]; AR [231,232,233]; HIF-1α [234]; PRMT5 [235]; PTEN [236]	AR activity [227]; mitochondrial dynamics, tumor chemotaxis, invasion, and metastasis in vivo [228]; anti-androgen resistance [229]; in vitro migration and invasion [230]; mitotic arrest [233]; potential role in PCa oncogenesis through PRMT5 [235]
UBE4A	Ubiquitin-protein ligase that probably functions as an E3 ligase; may also function as an E4 ligase complementing actions of another E3 ubiquitin ligase	Interleukin-like EMT inducer (ILEI) [237]	In vitro migration and invasion [237]
**HECT type**			
EDD	E3 ubiquitin-protein ligase, which is a component of the N-end rule pathway	Wnt/β-Catenin signaling [238]	Sensitivity of hormone-refractory PCa to docetaxel in vitro and in vivo [238]
E6AP	The founding member of the HECT (Homologous to E6AP Carboxyl Terminus) domain E3 ligases	NDRG1 [239], p27 [240]; PI3K, AKT [241,242], mTOR [241]	Acquisition of mesenchymal features, migration, ability for anchorage-independent growth [239]; tumor growth [240]; proliferation and invasion in bone metastasis [241]; cell growth, proliferation, apoptosis [242]; cellular senescence in vivo, radiation-induced cell death [243]
HACE1	HECT domain and ankyrin repeat-containing ubiquitin ligase		HACE1 is a critical chromosome 6q21 tumor suppressor involved in prostate cancer [244]
HECTD4	Probable HECT domain E3 ubiquitin-protein ligase	AR, MYC [138]	Tumorigenicity of AR-positive PCa cells [138]
HUWE1	WWE domain-containing protein 1, E3 ubiquitin protein ligase	HK2 [245]; c-MYC [246,247]	Metabolism and cancer stem cell expansion [245]; survival [246]; proliferation [246,247] and migration in vitro, and explant growth in vivo [247]
ITCH/AIP4	HECT-type E3 ubiquitin transferase Itchy homolog	ErbB3 [248]	ErbB3 ubiquitination and degradation in cancer cells through JNK1/2-dependent ITCH/AIP4 activation [248]
Nedd4	Comprised of a catalytic C-terminal HECT domain and N-terminal C2 domain and WW domains responsible for cellular localization and substrate recognition	IRS-2 [249]; AR [250]; ErbB3 levels and signaling [251]	IGF signaling and mitogenic activity [249]; cancer cell proliferation in vitro and in vivo; sensitization of cancer cells for growth inhibition by an anti-ErbB3 antibody [251]
SMURF1	SMAD specific E3 ubiquitin protein ligase 1	PTEN [252]	PCa progression [252]; invasion [253]
WWP1	WW domain-containing E3 ubiquitin protein ligase-1	TGFβ [254]; p63 [255]; KLF5 [256]	Migration and invasion [257]; 22Rv1 cells colony formation; PC-3 cells proliferation and TGFβ-mediated growth inhibition [254]; apoptosis [255]
WWP2	WW Domain Containing E3 Ubiquitin Protein Ligase 2	SUMO1-modified PTEN [258]	PCa development [258]

**Table 4:** Roles of (de-)acetylating enzymes in prostate cancer.

Enzyme	Involvement(s) in Prostate Cancer	Ref.
**KATs**		
**KAT2A**	KAT2A inhibition prevents interleukin (IL) 6-induced PCa metastases through PI3K/PTEN/AKT signaling by inactivating Egr-1	[307]
	Association between AR and histone acetyltransferase KAT2A increases histone H3 acetylation level on cis-regulatory elements of AR target genes	[308]
**KAT2B**	Promotes PKM2 acetylation and decreases PKM2 protein level through degradation through chaperone-mediated autophagy; promotes tumor growth	[309]
**CBP** **(KAT3A)**	CBP loss cooperates with PTEN haploinsufficiency to drive PCa	[310]
**p300 (KAT3B)**	p300-mediated acetylation of histone demethylase JMJD1A prevents its degradation by CHIP and enhances its activity	[227]
	p300/CBP inhibition enhances the efficacy of programmed death-ligand 1 blockade treatment	[311]
	Therapeutic targeting of the CBP/p300 bromodomain blocks the growth of CRPC	[312]
	p300 regulates fatty acid synthase expression, lipid metabolism and PCa growth	[313]
	p300 regulates AR degradation and PTEN-deficient prostate tumorigenesis	[314]
	The assembly of a macromolecular complex involving CBP/p300 results in acetylation of p53 at K373, a critical PTM required for its biological activity	[315]
	SKP2 is acetylated by p300 at K68 and K71, which promotes its cytoplasmic retention, and cytoplasmic SKP2 enhances cellular migration through ubiquitination and destruction of E-cadherin	[202]
	p300 is the dominant coregulator of the CBP/p300 pair for androgen-regulated gene expression in C4-2B cells; p300 is required at an early stage of chromatin remodeling and transcription complex assembly after binding of AR to the gene but before many critical histone modifications occur	[316]
	Function in the survival and invasion pathways of PCa cell lines	[317]
	p300 and CBP stimulate estrogen receptor-beta (ER-β) signaling and regulate cellular events in PCa	[318]
	IL-4 activates AR through enhanced expression of CBP/p300 and its histone acetyltransferase activity	[319]
	p300 modulates nuclear morphology in PCa and is required for androgen depletion independent activation of the AR	[320]
	p300 mediates STAT3 acetylation on Lys685, which mediates STAT3 dimerization and is reversible by type I HDAC	[321]
	CBP/p300 is a component of a transcriptional complex that regulates SRC-dependent hypoxia-induced expression of VEGF	[322]
	The downregulation of p300 inhibits PCa cell proliferation both at the basal level and on IL6 stimulation	[323]
	p300 mediates androgen-independent transactivation of the AR by IL6	[324]
	p300 and p300/CBP acetylate the AR at sites governing hormone-dependent transactivation	[325]
**Tip60** **(KAT5)**	Negatively regulates the proliferation of LNCaP cells via the caspase 3-dependent apoptosis pathway	[326]
	Associated with resistance to X-ray irradiation	[327]
	Inhibition by TH1834 increases the effect of ionizing radiation in PC-3 and DU145 cells, induces apoptosis and increases unrepaired DNA damage	[328]
	Interacts with ER-β to regulate endogenous gene expression such as CXCL12 and cyclin D2	[329,330]
	KAT5 and KAT6B positively regulate cell proliferation through PI3K/AKT signaling	[331]
	Inhibition by NU9056 induces a decrease of AR, PSA, p21 and p53 levels in LNCaP cells, which might explain the increase of apoptosis and the decrease of proliferation	[332]
	Overexpression increases the acetylation of the AR and its localization in the nucleus and promotes cell proliferation	[333]
	Tip60 and β-catenin complexes regulate expression of metastasis suppressor gene KAI1	[334]
	A possible role for Tip60 in the molecular pathway leading to the development of androgen-independent PCa following long-term androgen deprivation therapy	[335]
	Tip60 and HDAC1 regulate AR activity through changes to the acetylation status of the receptor	[336]
**MYST1** **(KAT8)**	Regulates androgen signaling in PCa cells	[337]
	Regulates NF-κB and AR functions during proliferation of PCa cells	[338]
	FOXP3 induces H4K16 acetylation and H3K4 trimethylation and activation of multiple genes by recruiting KAT8 and causing displacement of PLU-1	[339]
**KDACs**		
**Class I**	Maspin induction is a critical epigenetic event altered by class I HDACs in the restoration of balance to delay proliferation and migration ability of PCa cells	[340]
**HDAC1**	KLF5 inhibits STAT3 activity and tumor metastasis in PCa by suppressing IGF1 transcription cooperatively with HDAC1	[341]
	Involved in E-cadherin expression in PCa cells	[342]
	Ubiquitination of the AR and HDAC1 may constitute an additional mechanism for regulating AR function; HDAC1 and MDM2 function co-operatively to reduce AR mediated transcription that is attenuated by the HAT activity of the AR co-activator Tip60	[343]
**HDAC3**	Genetic knockdown of either HDAC1 or HDAC3 can suppress expression of AR-regulated genes, recapitulating the effect of HDAC inhibitor treatment	[344]
**HDAC4**	Positive regulator of AR SUMOylation, revealing a deacetylase-independent mechanism of HDAC action in PCa cells	[345]
	Recruitment of HDAC4 by transcription factor YY1 represses HOXB13 to affect cell growth in AR-negative PCa	[346]
**HDAC6**	Synergistic interaction with MEK-inhibitors in CRPC cells	[347]
	Metastatic prostate cancer-associated p62 inhibits autophagy flux and promotes EMT by sustaining the level of HDAC6	[348]
	Regulates AR hypersensitivity and nuclear localization via modulating Hsp90 acetylation in CRPC	[349]
**HDAC7**	HDAC7 localizes to the mitochondrial inner membrane space of prostate epithelial cells and exhibits cytoplasmic relocalization in response to initiation of the apoptotic cascade, which highlights a link between HDACs, mitochondria, and programmed cell death	[350]
**HDAC11**	HDAC11 depletion is sufficient to cause cell death and to inhibit metabolic activity in PC-3 cells	[351]
**SIRT1**	Modulates the sensitivity of PCa cells to vesicular stomatitis virus oncolysis	[352]
	Mesenchymal stem cells overexpressing SIRT1 inhibit PCa growth by recruiting NK cells and macrophages	[353]
	Loss of miR-449a in ERG-associated PCa promotes the invasive phenotype by inducing SIRT1	[354]
	SIRT1 and LSD1 competitively regulate KU70 functions in DNA repair and mutation acquisition	[355]
	The silencing of SIRT1 gene in PC-3 cells suppresses the movement, migration, and invasion, possibly via reversing the EMT process	[356]
	Loss of Sirt1 promotes prostatic intraepithelial neoplasia, reduces mitophagy, and delays Park2 translocation to mitochondria	[226]
	Existence of SIRT1 and MPP8 crosstalk in E-cadherin gene silencing and EMT	[357]
	Regulation of histone H2A.Z expression is mediated by SIRT1 in PCa	[358]
	Enhances matrix metalloproteinase-2 expression and tumor cell invasion of PCa cells	[359]
	SIRT1 induces EMT by cooperating with EMT transcription factors and enhances PCa cell migration and metastasis	[360]
	Inhibition of cortactin and SIRT1 expression attenuates migration and invasion of DU145 cells	[361]
	Deacetylation of FOXO3 by SIRT1 or SIRT2 leads to SKP2-mediated FOXO3 ubiquitination and degradation	[201]
	Disruption of a SIRT1-dependent autophagy checkpoint in the prostate results in prostatic intraepithelial neoplasia lesion formation	[362]
	Inhibition of SIRT1 activity increases the chemosensitivity of androgen-refractory PCa cells	[363]
	SIRT1 inhibition at the activity level as well as via shRNA results in a significant inhibition in the growth and viability of human PCa cells; inhibition of SIRT1 causes an increase in FOXO1 acetylation and transcriptional activation in PCa cells	[364]
	SIRT1 inhibition causes a decrease in cell growth, cell viability and the colony formation ability and an increase in FOXO1 acetylation and subsequent transcriptional activation regardless of p53 status; SIRT1 inhibition results in an increase in senescence in PC-3-p53 (wild type p53) cells whereas it results in an increase in apoptosis in PC-3 (lack p53) cells	[365]
	Upregulation of SIRT1 expression may play an important role in promoting cell growth and chemoresistance in androgen-refractory PC-3 and DU145 cells	[366]
	Required for antagonist-induced transcriptional repression of androgen-responsive genes by the AR	[367]
	SIRT1 is a regulator of AR expression and function	[368]
	FOXO1 activity in PCa cells is inhibited by deacetylation by SIRT1	[369]
**SIRT2**	Dysregulation of SIRT2 and histone H3K18 acetylation pathways associates with adverse PCa outcomes	[370]
**SIRT3**	Transcriptional repression of SIRT3 potentiates mitochondrial aconitase activation to drive aggressive PCa to the bone	[371]
	SIRT3 and SIRT6 promote PCa progression by inhibiting necroptosis-mediated innate immune response	[372]
	Inhibits PCa metastasis through regulation of FOXO3A by suppressing Wnt/β-catenin pathway	[373]
	Inhibits PCa by destabilizing c-MYC through regulation of the PI3K/AKT pathway	[374]
	Inactivation of SIRT3 leads to elevated SKP2 acetylation, which leads to increased SKP2 stability through impairment of the CDH1-mediated proteolysis pathway resulting in increase of SKP2 oncogenic function; cells expressing an acetylation-mimetic mutant display enhanced cellular proliferation and tumorigenesis in vivo	[202]
**SIRT4**	Mitochondrial PAK6 inhibits PCa cell apoptosis via the PAK6-SIRT4-ANT2 complex	[375]
**SIRT5**	SIRT 5 regulates the proliferation, invasion, and migration of PCa cells through acetyl-CoA acetyltransferase 1	[376]
**SIRT6**	E2F1 enhances glycolysis through suppressing Sirt6 transcription in cancer cells	[377]
	Inhibition of SIRT6 reduces cell viability and increases sensitivity to chemotherapeutics	[378]
**SIRT7**	SIRT7 depletion inhibits cell proliferation and androgen-induced autophagy by suppressing the AR signaling in PCa	[379]
	Promotes PCa cell aggressiveness and chemoresistance	[380]
	SIRT7 inactivation reverses metastatic phenotypes	[381]
